# Need, Enabling, Predisposing, and Behavioral Determinants of Access to Preventative Care in Argentina: Analysis of the National Survey of Risk Factors

**DOI:** 10.1371/journal.pone.0045053

**Published:** 2012-09-12

**Authors:** Eiman Jahangir, Vilma Irazola, Adolfo Rubinstein

**Affiliations:** 1 Institute for Clinical Effectiveness and Health Policy, Buenos Aires, Argentina; 2 Department of Cardiovascular Medicine, Vanderbilt University Medical Center, Nashville, Tennessee, United States of America; Fundación para la Prevención y el Control de las Enfermedades Crónicas No Transmisibles en América Latina (FunPRECAL), Argentina

## Abstract

**Introduction:**

Health care utilization is an important step to disease management, providing opportunities for prevention and treatment. Anderson’s Health Behavior Model has defined utilization by need, predisposing, and enabling determinants. We hypothesize that need, predisposing, and enabling, highlighting behavioral factors are associated with utilization in Argentina.

**Methods:**

We performed a logistic regression analysis of the 2005 and 2009 Argentinean Survey of Risk Factors, a cohort of 41,392 and 34,732 individuals, to explore the association between need, enabling, predisposing, and behavioral factors to blood pressure measurement in the last year.

**Results:**

In the 2005 cohort, blood pressure measurement was associated with perception of health, insurance coverage, basic needs met, and income. Additionally, female sex, civil state, household type, older age groups, education, and alcohol use were associated with utilization. The 2009 cohort showed similar associations with only minor differences between the models.

**Conclusions:**

We explored the association between utilization of clinical preventive services with need, enabling, predisposing, and behavioral factors. While predisposing and need determinants are associated with utilization, enabling factors such as insurance coverage provides an area for public intervention. These are important findings where policies should be focused to improve utilization of preventive services in Argentina.

## Introduction

Healthcare, like all other markets, consists of a need, demand, and supply of the product. Need in health care, is the capacity to benefit from the care. This concept incorporates wider social and environmental determinants of health; such as deprivation, housing, diet, education, and employment. [1] Demand is what the patients ask for and are the typical items that most physicians encounter. Finally, when supply meets that demand, actual health care is provided, which in turn can be measured through the utilization of health care services. [1] In a population, health care utilization is an important marker of access to and coverage of health services. The level of utilization varies within a population, differing amongst various social groups or people with different behaviors. [2]–[5].

The Andersen’s Behavioral Model of Health Care Utilization, initially developed in the late 1960’, suggests that people’s use of health services is a function of their predisposition to use services, factors which enable or impede use, and their need for care, thus providing a way to conceptualize these variations in utilization rates and consumption of medical resources. [6], [7] In this model, use of services is defined as a function of 3 main elements: need, enabling, and predisposing factors. Need factors, which have been shown to account for the majority of the explained variability in physician use, include the individual’s perceived health care need and other indicators of their health status. Factors such as self-reported number of symptoms, self-perceived health, number of bed days, restricted activity, and activities of daily living are part of the patient’s perceived need of health care. Enabling factors include items such as the individual’s income, health insurance status, and access to a source of regular care. Finally, predisposing factors include demographic variables, socioeconomic status, attitudes, and beliefs. [8]–[10]. Even though this model could either explain or predict use of services, and this is a matter of some debate, predisposing factors might be exogenous and enabling resources are necessary but not sufficient. In this regard, assuming the presence of predisposing and enabling conditions, the subject must perceive illness as a need for the utilization of health services. Perceived health may include different dimensions such as overall quality of life, perceived health, activities of daily living (ADL), depression, psychosocial distress and other psychological variables were among the strongest predictors of hospitalizations and physician visits. [11].

Andersen and Newman’s model of health care utilization has been mainly used for explaining health care utilization patterns by the general population. [12]–[17] Multiple studies have evaluated these determinants, describing both prior physician utilization as a strong predictor of subsequent physician use and items such as low-income status and a lack of motivation regarding prevention to health care procrastination. [18], [19] Additionally, differences in health care utilization exist amongst various social classes. [20] These findings do not only hold true for developed countries, but also for developing countries. [21]–[24].

Under this theoretical framework, we decided to use blood pressure measurement over the last year as an indirect marker for clinical preventive service utilization. The rational of this proxy is that blood pressure assessment is an integral part of clinical practice and the benefits of screening for hypertension in adults older than 18 years old are well established. [25], [26] Although evidence is lacking on the recommended optimal interval for screening adults for hypertension, most groups recommend measuring blood pressure yearly in normotensives, while also encouraging a check on every physician visit. [27]–[29] According to this premise, most individuals who make use of health services should have their blood pressure checked at some time in the process. In fact, 97.5% of the population in Buenos Aires has had their blood pressure measured at least once previously. [30].

The healthcare system in Argentina has three sectors: public, social security, and private. The public health system, covering 35% of the population, includes the national and provincial ministries as well as the network of public hospitals and primary health care centers which provide care to poor and uninsured persons. It is mainly financed by taxes. Social security, consisting of more than 300 different health funds mostly linked to trade unions, covers workers of the formal economy and their families, and is financed by payroll contributions of workers and employers. This sector provides health coverage to more than 50% of the population. Finally, the private sector is funded through direct and voluntary prepayments by insured members. In Argentina, the percentage of uninsured varies across the provinces ranging from 47% in Jujuy to 7.5% in Buenos Aires. [31] Health care in uninsured individuals relies solely on the public network of health care centers and hospitals. Like many other countries in Latin America, Argentina has major healthcare problems related to both equity and efficiency. Regarding equity, in healthcare insurance, there is a marked income gradient in insurance coverage, where more than 60 percent of the poorer 20 percent of the population has no insurance as compared to less than 10 percent in the wealthier 20 percent. In the tax-funded public system, hospital and ambulatory services are generally free at the point of care and delivered on demand, with a large variation in the complexity and the quality of services according to each district, where wealthier provinces have better quality services than poorer ones. Essential pharmaceuticals are included in a positive list delivered to all public of the primary care centers; more than 6,000, through a country-wide program (Program “Remediar”). For the Social Security sector, there is a compulsory package of benefits (PMO) that all funds are obliged to guarantee its coverage to their beneficiaries. Ambulatory drugs are subsidized in a proportion depending on the condition treated and may vary from 40% (some acute conditions) to 100% of a reference price. Since 2004, the coverage of most drugs for chronic conditions was increased to 70% to 100% of a reference price. There are no co-payments for the use of preventative services for both insured and uninsured populations. [32].

In this study, we hypothesize that need, enabling, predisposing -including and highlighting behavioral factors, are associated with utilization of clinical preventive services. Health beliefs are attitudes, judgment values and knowledge that people have about health. Considering that there is strong evidence on the correlation between use of preventative services and health beliefs, we decided to underscore behavioral risk factors in order to highlight their independent contribution as explanatory variables of use of health services. [33] By exploring these relationships, we hope to identify potential areas for intervention to improve the utilization of preventive services in Argentina.

## Methods

### Design

This study explores factors associated with health care access and utilization through secondary analysis of data obtained from the first wave (2005) and second wave (2009) of the Argentinean National Risk Factor Survey. These surveys are cross-sectional studies, repeated over time as part of a national surveillance system. The response rate was 86.7% in 2005, and 79.8% in 2009. [34].

Both surveys were obtained through anonymous forms that do not contain identifiable or potentially identifiable information. Additionally, this study does not involve merging these databases in such a way that individuals might be identified. According to national regulations, the data obtained from these surveys are public sources with unrestricted access and do not require informed consent from participants. The data sets have been made public by the Ministry of Health for research purposes and therefore, IRB review was not required for this study. No significant changes in the health care system, context and environment, occurred in Argentina between both national surveys.

### Population

The Argentinean National Risk Factor Survey is a nationally representative survey that included 41,392 participants in 2005 and 34,732 participants in 2009 from all districts of the country, sampled through a probabilistic multi-stage process. The surveys were based on a complex sample design and system of weighting that allowed computing population-based estimates of health conditions and behaviors. The prevalence of behavioral and socioeconomic factors were self-reported by participants during in-person interviews.

### Variables

The primary outcome for this study was utilization of clinical preventive health services, defined as having at least one blood pressure check within a year from the survey.

Exposures were taken from the first wave of the National Risk Factor Survey (FNRFS-2005), considering potential need, enabling, predisposing, and behavioral risk factors. Need factors included general perceived health measured by a single question on health perception from the SF-36 survey, a widespread questionnaire used to measure health-related quality of life, with categories defined as bad/regular, good, and very good/excellent. [35], [36] We also included the current state of health according to the EQ-5D (Euroqol) categories (no limitations versus moderate or severe limitations, in five dimensions: mobility, self-care, usual activities, pain or discomfort, and anxiety or depression). This tool, is used to measure health utilities and social preferences. Both questionnaires were included in both surveys, and validated in Argentina by our group. [37], [38] Enabling factors included insurance status (Private, social, or public coverage versus no coverage), employment (yes/no), basic needs met (all basic needs met versus ≥1 basic need unmet; determined as an aggregate score consisting of inadequate living, overcrowded household, living in a household without a bathroom, or having school- age children who are not attending school), and household income (lowest 40% bracket versus middle-high 60% bracket). Predisposing factors included characteristics such as sex, civil state (married or joined versus separated, widowed, divorced, or single); household situation (single or separated household with/without other members present versus married or joined household with/without other members present), age (18–34 years old, 35–49 years old, 50–64 years old, and ≥65 years old), and education (primary education incomplete versus primary education complete or more). As said above, we analyzed separately behavioral risk factors from other predisposing factors in the explanatory models. The behavioral factors that were measured in both 2005 and 2009 national risk factor surveys included level of physical activity (low activity versus moderate or intense activity), current tobacco use (any), heavy drinking (consumption of more than one drink per day for women, or more than two drinks per day for men, weighted average), and any daily fruit or vegetable consumption (at least one portion). Physical activity was measured using the International Physical Activity Questionnaire (IPAQ), included in the surveys. Smoking status, alcohol abuse, and fruit or vegetable consumption were measured using standardized questions from the WHO STEPwise approach to Surveillance (STEPS). [39] The prevalence of these exposures in the participant pool can be seen in Table 1.

**Table 1:** Prevalence of need, enabling, predisposing, and behavioral factors in the Argentinean Survey of Risk Factors.

	ENFR 2005	ENFR 2009
	(n = 41,392)*	(n = 34,732)*
Age, mean (SD)	43.92 (17.66)	44.57 (17.85)
	**Number (%)**	**Number (%)**
**Need factors**		
General perceived health- bad/regular	9,403 (22.7)	7,362 (21.20)
General perceived health- good	18,173 (43.9)	15,138 (43.59)
General perceived health- verygood/excellent	13,816 (33.38)	12,232 (35.22)
**Enabling factors**		
Insured	27,194 (66.49)	24,431 (74.41)
Employed	26,174 (63.23)	21,560 (62.08)
All basic needs met	35,055 (84.69)	29,689 (85.48)
Income- low 40%	15,596 (41.08)†	13,696 (43.09)‡
Income- mid-high 60%	22,3711 (58.92)†	18,086 (56.91)‡
**Predisposing factors**		
Male	17,827 (43.1)	15,028 (43.27)
Married or joined	22,501 (54.36)	19,019 (54.76)
Household- marriedor multiperson§	27,574 (66.62)	22,748 (65.50)
Age 18–34 years old	15,016 (36.28)	12,338 (35.52)
Age 35–49 years old	11,714 (28.30)	9,577 (27.57)
Age 50–64 years old	8,267 (19.97)	7,066 (20.34)
Age ≥65 years old	6,395 (15.45)	5,751 (16.56)
Without formal instruction	847 (2.05)	699 (2.01)
Primary school complete or incomplete	14,644 (35.38)	11,693 (33.67)
Secondary School complete or incomplete	15,002 (36.24)	13,374 (38.51)
More than secondary school	10,899 (26.33)	8,966 (25.81)
Moderate/intense physical activity#	22,044 (53.16)	15,143 (43.60)
Current tobacco use, any#	12,651 (30.56)	9,214 (26.53)
Heavy drinking#	4,802 (11.6)	4,136 (11.91)
Any daily fruit/vegetableconsumption#	28,004 (67.66)	21,560 (62.08)

* Total responses unless otherwise noted.

† Total responses = 37,967.

‡ Total responses = 31,782.

§ Living with or without other members.

# Behavioral risk factors.

### Data Analysis

Data analysis was undertaken following a two-stage approach to study the relationship between need, enabling, predisposing (including behavioral factors), and health care access and utilization of clinical preventive services.

In the first stage, using data from the FNRFS-2005, we fitted bivariate logistic regression models with one covariate at a time. Those variables that were statically significant (p value <0.10) were then included in a multivariable logistic model and tested for significance and confounding effects. The joint significance of the variables that were not selected in the first place was also explored. Finally, first-degree interaction terms were tested on the main model. In the second stage, we ran the model obtained in the first stage using data from the second wave of the National Risk Factor Survey (SNRFS-2009), and compared results in order to validate our findings. Statistical analysis was performed using Stata Statistical Software version 11.0 (Stata Corp, College Station, Texas).

## Results

### First Stage

Data from the FNRFS-2005, showed that measurement of blood pressure in the last year was significantly associated with need factors including bad/regular or good perceived health versus very good/excellent; enabling factors including having insurance coverage, having all basic needs met, and living in the 60% middle-high income bracket; predisposing factors including female sex, married or joined civil state, living in a single, separated, widowed, or divorced household, older age groups, completing at least primary school; and the behavioral factor such as not being a heavy drinker. Employment status and tobacco use were retained in the model because of a confounding effect. (Table 2) Age ≥65 years old [OR 3.83 (95% CI 3.46–4.24)], age 50–64 years old [OR 2.17 (95% CI 2.03–2.33)], and bad/regular perceived health [OR 2.33 (95% CI 2.16-2.5)] had the highest association with participants’ measurement of blood pressure in the last year.

**Table 2:** Association between need, enabling, predisposing, and behavioral factors with blood pressure measurement over past year. Results from the multivariate logistic regression analysis.

	ENFR 2005	ENFR 2009
	Measured- 28,582	Measured- 24,605
	Not measured- 12,810	Not measured- 9,999
**Need Factors**		
General perceived health* - good	1.28 (1.21–1.35)	1.22 (1.14–1.30)
General perceived health* - bad/regular	2.33 (2.16–2.5)	2.18 (1.97–2.42)
**Enabling Factors**		
Insured	1.6 (1.52–1.69)	1.40 (1.30–1.51)
Employed	1.02 (0.97–1.08)^§§§^	0.98 (0.91–1.06)^§§§^
All basic needs met†	1.26 (1.18–1.34)	1.15 (1.05–1.26)
Income‡	1.19 (1.12–1.26)	1.10(1.03–1.18)
**Predisposing Factors**		
Male	0.59 (0.56–0.62)	0.81 (0.75–0.86)
Married or joined§	1.32 (1.24–1.41)	0.97 (0.89–1.06)^§§§^
Household- Married or multiperson* *	0.81 (0.76–0.87)	1.12 (1.02–1.24)
Age††−35–49	–	1.22 (1.13–1.32)
Age††−50–64	2.17 (2.03–2.33)	2.18 (1.98–2.40)
Age††−≥65	3.83 (3.46–4.24)	3.85 (3.37–4.41)
Primary school completed or more‡‡	1.2 (1.11–1.3)	1.12 (1–1.25)*
Physical activity§§,#	–	0.93 (0.87–0.99)
Tobacco*** ^,^ #	0.96 (0.91–1.01)^§§§^	0.84 (0.79–0.90)
Alcohol†††, #	0.78 (0.73–0.84)	0.74 (0.67–0.81)
Any daily fruit/vegetable consumption#	1.26 (1.18–1.34)	1.22 (1.10–1.34)

* Versus very good/excellent (SF-36).

† Versus 1 or more basic needs not met.

‡ Middle-high (60%) vs low (40%).

§ Versus single, separated, widowed, or divorced.

** Versus single or separated household with or without other members.

†† Versus 18–34 years old age group.

‡‡ Versus primary school not completed.

§§ Moderate/intense versus low activity.

# Behavioral risk factors.

*** Any current use.

††† Alcohol abuse, total weighted average.

All p values significant to <0.05 unless noted by^§§§^.

Data presented as odds ratio (95% Confidence intervals).

### Second Stage

After running the model built in the first stage, using the dataset of the SNRFS-2009, we found that most of the variables were retained as factors significantly associated with measurement of blood pressure in the last year. After adjusting for employment status, civil state, and education; need factors included perceived health; while enabling factors included insurance coverage, all basic needs met, and living in the 60% middle-high income bracket. Interactions with sex and age were not significant. Predisposing factors for blood pressure measurement were female sex, living in a married or multiperson household, and older age groups. Finally, behavioral factors included abstaining from tobacco use and not being a heavy drinker. (Table 2) Age ≥65 years old [OR 3.85 (95% CI 3.37–4.41)], age 50–64 years old [OR 2.18 (95% CI 1.98–2.40)], and bad/regular perceived health [OR 2.18 (95% CI 1.97–2.42)] again had the highest association with participants obtaining a blood pressure check in the last year. On the other hand, living in a multiperson household had only a borderline effect [OR 1.12 (CI 1.02–1.24)] in the second model.

While most associations described in the 2005 and 2009 data were concordant in direction and magnitude, there were some notable differences between the two models. These differences occurred amongst civil state, household type, education, and tobacco use. Of these, tobacco use and education demonstrated statistically significant associations in one sample but not in the other while maintaining similar directions of association. Civil state and household type demonstrated opposite associations for health care utilization. (Table 2) It is unclear why these associations differed between the two surveys, but it should be explored in more detail in further waves of the survey.

## Discussion

In this study we set forth to explore the determinants of utilization to preventive services in Argentina using the Andersen’s Behavioral Model of Health Care Utilization highlighting some behavioral factors included in the surveys. Using data from both the first (2005) and second (2009) wave of the Argentinean National Risk Factor Survey, we are the first to evaluate utilization of preventive services in a broad and representative Argentine population. Additionally, using both data sets, we are able to demonstrate the stability and robustness of our estimates and model.

Through this analysis we are able to describe a number of interesting associations. First, we have demonstrated that Anderson’s Health Behavior Model of Health Care Utilization holds true in an Argentinean population. Need, enabling, and predisposing factors, including behavioral factors, all are significantly associated with access to preventative services using blood pressure measurement over the last year as a surrogate marker. Amongst these determinants, increasing age and a perception of poor health had the largest associations with utilization. (Table 2) While it may be intuitive that older individuals will seek regular medical visits, it is concerning that middle-aged adults (age 35–49), arguably the most productive subgroup of the population and who remain susceptible to hypertension, diabetes, and a host of other diseases, do not appear to be utilizing preventive services in Argentina. Further research into how to increase preventive care in this important subgroup is needed. Additionally, the relationship between perceived health status and health care utilization remains unclear due to the complexities associated between these items. It can be speculated that individuals who need more frequent medical care may perceive their health status as worse due to the frequent use of medical services. On the other hand, individuals who have a poor perception of their own health may seek medical care more frequently. Therefore, it is difficult to assess causation and due to the cross-sectional nature of this study we cannot infer causality between perception and utilization. This is an interesting association and an area that should be assessed more fully in the future.

In this analysis, enabling and behavioral variables, among the predisposing factors, were also associated with utilization of clinical preventive services. For instance, having health insurance, having all basic needs met, and earning in the 60% high income bracket were all associated with increased utilization of preventative services while employment status had no effect. Of these, health insurance coverage had the highest association with having a blood pressure check in the last year. (Table 2) This implies that a lack of insurance coverage may serve as a large barrier to utilization. Therefore, by providing health care coverage to the population, we may be able to increase use of preventative services. Finally, this study also demonstrated that certain behavioral factors are associated with utilization. The most consistent findings were that heavy drinkers were less likely to have their blood pressure checked, while individuals who consumed fruits or vegetables regularly had their blood pressure measured more frequently. These associations, while complex and multifactorial, may be due to the value system and beliefs placed on participant’s individual health as opposed to an issue of access.

While this is the first study in Argentina to fully evaluate the association between need, enabling, predisposing, and behavioral factors to utilization of preventive health services in a large population, similar studies on other countries have been reported. For instance, Ham et al. evaluated the predictors of making contact with a health care service among newly diagnosed hypertensive individuals in South Korea. [40] They described similar predisposing and behavioral determinants as our study, including age, sex, alcohol consumption, and tobacco use. [40] Another study described older age and employed status as predictors of adherence to mammography guidelines in a rural population of the US. [41] Interestingly, this study described the association of employment status, but not health insurance, as a predictor to adherence. This is a finding that differs with what we report here; mainly that insurance coverage and not employment status was an important determinant of utilization. Nevertheless, insurance and employment status may be highly correlated, as is the case both in Argentina and the US. Additionally, there have been a few studies in Latin America evaluating predictors of health care utilization. One study, in Colombia, described that differences in health service use was related to differences in social, economic and political status. [21] Other studies emphasize the strong association between health insurance coverage and improved use of preventative services, even when accounting for other need, enabling, and predisposing predictors. [42]–[44] The similarity amongst all these studies remains that need, enabling, and predisposing factors, including behavioral determinants, and especially access to health insurance, appear to have strong association with utilization of health care.

While we have reported some interesting findings, our study has some limitations. First, our analysis used self-reported responses, as obtained in the surveys, with no confirmation of blood pressure measurement from the medical records. Using self-reported responses may lead to information bias that could affect our results, though this is unlikely provided that all data came from a population-based survey. Next, while we used blood pressure checks as a general proxy of access and utilization of clinical preventive services due to its availability and simplicity, other preventives services that were not sought in our analysis could have shown different predictors, such as screening for cervical or breast cancer. Unlike these early diagnoses preventive services, regular blood pressure checks are a universal preventive service involving all adults of both sexes, regardless of any particular risk exposure. Due to the cross-sectional nature of the surveys, all information on need, predisposing, enabling, and behavioral factors were collected simultaneously with data regarding our outcome, blood pressure measurement in the last year. This lack of a temporal collection could limit the ability of the models to infer causality.

Finally, it is important to recognize that the determinants of preventive services, as any other health service, must be analyzed taking into account the particular health system context in each country. In this regard, the extent of health coverage, the degree of financial protection, the content of the benefit package, and the provider payment schemes and incentives, might affect how preventive services are actually delivered. Although in theory preventive services are universally covered without co-payments in Argentina, including the public sector for the uninsured, in practice this vulnerable population deals with many barriers to access these services. The scope of this study does not allow us to explore these important factors involved in the utilization of health care services though.

### Conclusions

This study has explored important predictors of utilization of preventive services. From all of the associations described, the dimensions where public health policies may most effectively be targeted are the enabling factors, specifically providing universal health coverage. In this regard, it is dismaying that about 35% of the Argentine population still has no health insurance and relies solely on the public health network of each province or district. [32] These are important areas where public policy goals can be directed. Hopefully this study will lead to future, more detailed policy directed research to promote changes to reduce health disparities in Argentina.
